# Progesterone receptor B over progesterone receptor A prevents recurrence in bilateral endometriomas

**DOI:** 10.1590/1806-9282.20240485

**Published:** 2024-09-16

**Authors:** Ozgur Aslan, Sukru Yildiz, Cihan Kaya, Serdar Altinay, Ilke Esin Aydiner, Esra Karabulut, Murat Ekin, Levent Yasar

**Affiliations:** 1 Muş State Hospital, Department of Obstetrics and Gynecology – Muş, Turkey.; 2 University of Health Sciences Turkey, Bakirkoy Dr. Sadi Konuk Training and Research Hospital, Department of Obstetrics and Gynecology – İstanbul, Turkey.; 3 Acibadem Mehmet Ali Aydinlar University, Acibadem Bakirkoy Hospital, Department of Obstetrics and Gynecology – İstanbul, Turkey.; 4 University of Health Sciences Turkey, Bakirkoy Dr. Sadi Konuk Training and Research Hospital, Department of Pathology – İstanbul, Turkey.

**Keywords:** Endometriosis, Laparoscopy, Progesterone, Recurrence

## Abstract

**OBJECTIVE:**

Endometriosis is a disease in which stromal cells and endometrial glands extend outside of the uterine cavity. Nevertheless, treatment failure and recurrence cause difficulties in management. This study aimed to evaluate the receptor-level components of bilateral endometriomas in the recurrence state.

**METHODS:**

Our retrospective cohort study was conducted with patients who underwent surgery for bilateral endometriomas between 2015 and 2021. In total, 113 patients were allocated. A total of 76 patients did not meet the eligibility criteria, and the data of 37 patients were evaluated. Medical treatments, recurrences, and postoperative follow-up data were collected. In archived tissue samples, measurements of progesterone receptor A and progesterone receptor B, histoscores and immunoreactivity scores, and their ratios were calculated in the group that received no postoperative medical treatment. Criteria for recurrence were a repeat operation and/or the detection of a new endometrioma>2 cm at the follow-up examination.

**RESULTS:**

No recurrence was observed in 73.0% (n=27) of the cases, whereas recurrence was observed in 27.0% (n=10) of the participants. Patients without recurrence had significantly higher progesterone receptor B histoscore/progesterone receptor A histoscore and progesterone receptor B immunoreactivity score/progesterone receptor A immunoreactivity score results (p=0.01). Nevertheless, when the histoscores and immunoreactivity scores for both receptors were contrasted separately, there was no appreciable difference between them.

**CONCLUSION:**

The dominance of progesterone receptor B over progesterone receptor A was inversely proportional to the recurrence status in bilateral endometriomas. Furthermore, our study revealed that assessing receptor levels alone did not result in a significant difference in recurrence.

## INTRODUCTION

Endometriosis is defined as a benign, heterogeneous, estrogen-dependent, and progesterone-resistant disease. Endometriosis affects approximately 10% of reproductive-aged women^1^. The treatment of endometriosis requires experience, and the recurrence after primary surgery is around 15–50%. Frequently, dysmenorrhea affects women's academic and occupational performance. It significantly contributes to employee absenteeism and a decline in the overall quality of life^2,3^.

During the menstrual cycle, estrogen promotes epithelial proliferation to develop endometrial thickness, while progesterone inhibits estrogen-induced proliferation and allows stromal cells to initiate decidualization^4^. Therefore, resistance to uterine artery blood flow is diminished when progesterone hormone is secreted during the mid-luteal phase in the presence of estrogen^5^.

Progesterone receptor (PR) expression is altered in endometriotic lesions. A study by Tarumi et al. evaluating 132 patients undergoing surgery for endometriosis reported that the level of PR immunoreactivity in epithelial cells was significantly lower in relapsed cases than the control group^6^.

Variations at the receptor level are believed to play an important role in treatment resistance, and they contribute to the difficulty of finding a definitive therapy of the disease^6^. Progesterone exerts its action through intracellular PRs, such as progesterone receptor A (PRA) and progesterone receptor B (PRB). PRB has been shown to be a more potent activator of progesterone target genes than PRA, whereas PRA is a more potent repressor of PRB^6^.

The increased level of the PRB gene also known as an indicator of progesterone resistance in endometriosis^5^. Besides, promoter hypermethylation and microRNA dysregulation are accused as the possible pathways of PRB loss in endometriosis^7^. PRB expression was typically found to be decreased in endometriosis lesions or eutopic endometrium, according to studies that distinguished between the two. In contrast, reports regarding the PRA expression were inconsistent^8^.

The presence of bilateral endometriomas is known as the reason for a severe reduction in ovarian reserve and a high rate of recurrence, and it has been estimated that 28% of women with endometriosis have bilateral ovarian endometriotic cysts^9^. To date, there is no study evaluating PR levels and the rate of PRB/PRA in patients with bilateral endometriomas. Since bilateral endometriomas have detrimental effects on women in their reproductive years, it could be helpful to assess bilateral endometriomas at the receptor level during the primary surgeries to determine the recurrence rate.

This study aimed to assess the immune-histopathological PR scores of patients who underwent bilateral endometriomas.

## METHODS

This retrospective cohort study was conducted with 113 patients aged between 18 and 50 years who underwent surgery at our hospital for bilateral endometriomas between 2015 and 2021. The study was approved by the Clinical Research Ethics Committee of a Training and Research Hospital (approval number: 22-05-09). A written informed consent was obtained for the use of operating materials, with personal data being treated confidentially.

In addition to demographic data and laboratory test results, the size of the recurrent endometrioma after surgery, the need for perioperative hysterectomy, the presence of obliteration of the Douglas sac, and the preoperative medical treatment status (progesterone derivatives and gonadotropin-releasing hormone agonists) were recorded. A reoperation for endometriosis or the revealing of a new endometrioma>2 cm by transvaginal ultrasound examination 3 months after the initial surgery was defined as a recurrence^10^. Patients were examined at 3-month intervals, and the total follow-up period was set at 24 months^3,11,12^.

A total of 76 patients were excluded from the study because of concomitant malignancies (n=3), a lack of follow-up data (n=18), a lack of suitable specimens for immunohistochemistry (n=14), or hormonal suppression treatment occurring after the initial surgery (n=41). Since the pain from endometrioma appears to be related to periodic microbleeds within the endometriotic lesions, postoperative hormonal treatment can inhibit implant formation and alleviate endometriosis-related symptoms^13^.

Pathology specimens were obtained from 37 patients who met the study's eligibility criteria.

### Immune-histopathology analysis

Three-micrometer-thick tissue sections were placed on slides and dried overnight at 37°C. Subsequently, tissue slides were randomly selected from fully sectioned lesions. The sections were dewaxed in xylol, rehydrated using an ethanol solution, and incubated in an ethylene diamine tetra-acetic acid buffer for 11 min.

The sections were incubated for 20 min in 3% H_2_O_2_ at room temperature. To block nonspecific binding, sections were isolated from PRA RTU Bond 7 mL in the protein block (EnVision FLEX Mini Kit, High pH) for 10 min at room temperature and then overnight at 4°C. PRA C. Liq. 1 mL (1:100) and PRB Conc. 0.1 mL (1:800) were incubated. The mixture was then kept at room temperature for 30 min. Slides were counterstained with hematoxylin and dried.

Slides were divided into four groups according to the percentage of positively stained nuclei (PP) and classified as follows: Group 1 (low) had<25% staining, group 2 (moderate) had 25–50% staining, group 3 (high) had 50–75% staining, and group 4 (very high) had>75% staining^14^. Staining intensity (IS) was scored as follows: 1=mild, 2=moderate, and 3=high^14^. Following this, pathological specimens were randomly distributed and analyzed by an experienced pathologist (S. A). In addition, the immunoreactivity score (IRS) was obtained using the following formula: PP (1–4) × IS (1–4). Finally, the stromal IS was evaluated, and the slides were divided into the three following categories: 1=low (+), 2=moderate (++), and 3=high (+++)^14^.

Histoscores (H scores) for PRA and PRB were calculated as follows: H score=(3 × S)+(2 × M)+(1 × W) over S (percentage of strong staining), M (percentage of moderate staining), and W (percentage of weak staining)^15^. The PRB H score, PR H score ratio (PRB H score/PRA H score), PRB IRS, and PR IRS ratio (PRB IRS/PRA IRS) were recorded. Both scoring systems (H score and IRS) were used to confirm whether there would be a difference in comparisons at the end of the study.

### Statistical analysis

For statistical analysis, the NCSS (Number Cruncher Statistical System) 2007 Statistical Software (NCSS LLC, Kaysville, Utah, USA) program was used. Descriptive statistical methods (mean, standard deviation, median, frequency, percentage, minimum, and maximum) were used to analyze the study data. The Mann-Whitney U test was used for comparisons between two groups of quantitative variables that did not show a normal distribution.

Fisher's exact test and the Fisher-Freeman-Halton test were used to compare qualitative data. The cutoff was determined by diagnostic screening tests and receiver operating characteristic (ROC) analysis for the PRB H score/PRA H score and PRB IRS/PRA IRS ratios. In multivariate analyses, logistic regression analysis was used to determine the risk factors affecting recurrence. Statistical significance was assumed to be p<0.05.

## RESULTS

The ages of the patients ranged from 20 to 48 years, with an average of 38.76±7.30 years. Body mass index (BMI) values varied between 16.3 and 32.8 kg/m^2^; the mean value was 24.76±3.15. In terms of parity, the women had a range of 0–4 and an average of 1.59±1.14.

While recurrence was not detected in 73% of cases (n=27), it was observed in 27% of cases (n=10). In eight cases (21.6%), concomitant hysterectomy procedures were performed. Douglas obliteration was observed in 21 patients (56.8%). In addition, 33 (89.1%) patients did not have preoperative medical treatment, 3 (8.1%) patients used progesterone derivatives, and 1 (2.7 %) patient had gonadotropin-releasing hormone agonist (GnRHa) therapy.

A comparison of the tissue samples from the recurrent and nonrecurrent groups showed that the tissue segment from the nonrecurrent cases had almost negligible PRA-specific staining. In contrast, the staining levels of PRA and PRB were nearly identical in the recurrent cases.

The PRB H score/PRA H score and PRB IRS/PRA IRS of patients without recurrence were significantly higher than those of cases with recurrence (p=0.001; p<0.01; Table 1). This significance was used to calculate the cutoff for the PRB H score/PRA H score and PRB IRS/PRA IRS. In contrast, the PRA and PRB H scores and the IRSs of the cases showed no statistically significant difference according to the relapse status (p>0.05). ROC analysis and diagnostic screening tests were used to determine the cutoff point according to the presence of recurrence (Table 2). For the 0.588 cutoff value of the PRB H score/PRA H score, the sensitivity was 80%, the specificity was 85.2%, the positive predictive value was 66.7%, the negative predictive value was 92%, and the accuracy was 83.8%. The area under the ROC curve obtained was 92.6%. Furthermore, for the cutoff value of 0.333 for the PRB IRS/PRA IRS, the sensitivity was 80%, the specificity was 74.1%, the positive predictive value was 53.3%, the negative predictive value was 90.9%, and the accuracy was 75.7%. The area under the ROC analysis obtained was 83.7%.

**Table 1:** Comparisons of the study group considering endometriosis recurrence.

		Recurrence		p
		Absent (n=27)	Present (n=10)	
Age	Med±Sd	37.78±8.11	41.40±3.50	a0.353
BMI (kg/m²)	Med±Sd	25.26±2.88	23.41±3.60	a0.353
Parity (n)	Median (min–max)	2 (0–4)	1.5 (0–3)	a0.533
Ca-125	Med±Sd	83.75±67.04	74.33±57.54	a0.984
Cyst size (cm)	Med±Sd	6.47±3.81	5.01±1.79	a 0.538
PRA H Score	Med±Sd	192.78±46.54	213.50±60.37	a0.271
PRB H Score	Med±Sd	141.67±35.16	105.00±60.59	a0.130
PRB H/PRA H	Med±Sd	0.75±0.16	0.45±0.24	a0.001**
PRA IRS	Med±Sd	0.59±3.91	8.40±3.37	a0.098
PRB IRS	Med±Sd	3.56±3.05	2.90±3.03	a0.408
PRB IRS/PRA IRS	Med±Sd	0.63±0.29	0.28±0.23	a0.001**
Hysterectomy	Absent	22 (81.5)	7 (70.0)	b0.655
	Present	5 (18.5)	3 (30.0)	
Douglas obliteration	Absent	13 (48.1)	3 (30.0)	b0.461
	Present	14 (51.9)	7 (70.0)	
Preoperative treatment	No treatment	23 (85.2)	10 (100.0)	c0.668
	Progesterone	3 (11.1)	0	
	GnRHa	1 (3.7)	0	

a Mann-Whitney U test.

b Fisher exact test.

c Fisher-Freeman-Halton test.

** p<0.01. Comparisons could not be made due to the lack of observation.

**Table 2:** Receiver operating characteristic analysis for PRB H/PRA H and PRB IRS/PRA IRS scores.

	Cutoff	Sensitivity/specificity	ppv	npv	auc	95%CI	p
PRBH/PRA H Ratio	≤0.588	80.0/85.2	66.7	92.0	0.926	0.8445–1.000	0.001*
PRBIRS/PRAIRS Ratio	≤0.333	80.0/74.1	53.3	90.9	0.837	0.685–0.989	0.002*

* p<0.01. ppv: positive predictive value, npv: negative predictive value, auc: area under curve. For the cutoff value of the PRB H/PRA H level of 0.588, sensitivity 80%, and specificity 85.2%; the positive predictive value is 66.7%, the negative predictive value is 92%, and the accuracy is 83.8%. The area under the ROC curve obtained was 92.6% with a standard deviation of 4.2%. For the cutoff value of PRB IRS/PRA IRS level of 0.333, sensitivity 80%, and specificity 74.1%, the positive predictive value is 53.3%, the negative predictive value is 90.9%, and the accuracy is 75.7%. The area under the ROC curve obtained was 83.7% with a standard deviation of 7.7%.

We conducted a logistic regression analysis using the data provided in Table 1, including age, endometrioma size, PRB H score/PRA H score, and PRB IRS/PRA IRS (Table 3). Accordingly, the PRB H score/PRA H score was found to be 17.669 (95% confidence interval [CI]: 1.62–192.56) times associated with endometriosis recurrence. The effects of other variables were not significant.

**Table 3:** Logistic regression analysis of risk factors affecting endometriosis recurrence.

	p	ODDS	95%CI	
			Lower	Upper
Age (year)	0.329	1.086	0.920	1.282
Endometrioma size (cm)	0.358	0.775	0.450	1.334
PRB H/PRA H≤0.588	0.018*	17.669	1.621	192.560
PRB IRS/PRA IRS≤0.333	0.251	3.826	0.388	37.731

* p<0.05. The PRB H score/PRA H score was found to be 17.669 (95% confidence interval [CI]: 1.62–192.56) times associated with endometriosis recurrence.

## DISCUSSION

This study showed that the PR-H score ratio (PRB H score/PRA H score) and the PR-IRS ratio (PRB IRS/PRA IRS) were negatively associated with the recurrence in patients with bilateral endometriomas.

Regarding the estrogen and PR levels in endometriosis patients, Brichant et al. published a study evaluating the results of 41 endometriosis patients^14^. In this study, medical treatment regimens were evaluated in four main groups: One group included 18 patients who received no medical treatment prior to surgery; the other three groups included 23 patients in total who used medications (combined oral contraceptives (COCs), progestins, and GnRH agonists) to treat endometriosis. Using the IRS system for both receptors, immunohistochemical scores were assigned to patients receiving or not receiving prior medical treatment. In particular, IRS values for PR-stained lesions were systematically lower in medication groups compared with untreated patients^14^. This novel study conclusively demonstrated that treated patients had lower PR IRSs.

Another study by Attia et al. showed that PRB mRNA and protein levels were significantly decreased in endometriotic lesions, although PRA isoforms were generally preserved^16^. Attia et al. also evaluated receptor levels using the western blot technique in 18 patients who underwent surgery for endometriosis-related symptoms but did not receive preoperative medications^16^. A negligible amount of the PRA isoform but no PRB isoform was detected in the endometriotic specimens. Comparing the PRB/PRA ratios in our study to those in the literature, we found that the results are consistent. Consequently, it can be deduced that the proportion of PR levels significantly influences the recurrence of postoperative diseases. In particular, it has been suggested that decreased PR levels induce PR. A low PR may explain why progestogen-containing medications are associated with treatment failure in certain patients^15,17^.

A strength of our work lies in the assessment of receptor status and the postoperative clinical course. Our work also represents the largest study comparing receptor-level testing and recurrence in patients with bilateral endometriomas. Another strength of our study includes the wide range of immunohistochemical assessment methods used. Using two scoring systems offered the advantage of confirming the study results while minimizing subject bias. The main disadvantage of our work is that, although a large patient population was studied, the number of cases that met the inclusion criteria was limited.

## CONCLUSION

Understanding the mechanisms of therapeutic success or failure is crucial for guiding clinical decisions and informing future research in this area. The development of new molecules for the medical treatment of endometriosis should aim at new strategies to overcome resistance mechanisms.

In summary, it was found that the PR-H score ratio and the PR-IRS ratio were negatively associated with the recurrence in patients with bilateral endometriomas. Besides, the PRB H score/PRA H score could be an independent determinant of recurrence. Future studies will allow us to determine whether the presence of PR heterogeneity after progestogen therapy leads to further PR-resistant diseases.
